# ^68^Ga-FAPI-04 PET/CT in the Diagnosis of Hepatocellular Carcinoma Associated with Cirrhosis: Diagnostic Value, Correlation Between PET Parameters of the Tumor and Its Size, and PIVKA-II Levels

**DOI:** 10.3390/diagnostics16020249

**Published:** 2026-01-13

**Authors:** Zhamilya Zholdybay, Zhanar Zhakenova, Bekzhan Issamatov, Madina Gabdullina, Yevgeniya Filippenko, Suriya Yessentayeva, Galymzhan Alisherov, Jandos Amankulov, Ildar Fakhradiyev

**Affiliations:** 1 “Visual Diagnostics” Department, Asfendiyarov Kazakh National Medical University, Almaty 050012, Kazakhstan; zholdybay.z@kaznmu.kz (Z.Z.); isamatov.b@kaznmu.kz (B.I.); gabdullina.m@kaznmu.kz (M.G.); filippenko.e@kaznmu.kz (Y.F.); or galymzhan.alisherov@hilife.kz (G.A.); zh.amankulov@kaznmu.kz (J.A.); fakhradiyev.i@kaznmu.kz (I.F.); 2 Oncology and Radiology Department, Kazakh-Russian Medical University, Almaty 050004, Kazakhstan; info@medkrmu.kz; 3 Department of Nuclear Medicine, Limited Liability Company “HiLife”, Almaty 050031, Kazakhstan; 4 Department of Radiology and Nuclear Medicine, Kazakh Institute of Oncology and Radiology, Almaty 050022, Kazakhstan; 5 College of Medicine, Korea University, Seoul 02841, Republic of Korea

**Keywords:** ^68^Ga-FAPI-04 PET/CT, hepatocellular carcinoma, liver cirrhosis, diagnostic performance, SUVmax, TBR, PIVKA-II

## Abstract

**Background/Objectives:** Hepatocellular carcinoma remains a major cause of death from cancer globally. While ^18^F-FDG PET/CT is commonly used for tumor imaging, its sensitivity is limited, especially due to high liver background uptake. Recently, ^68^Ga-FAPI PET/CT, which targets fibroblast activation protein in tumor stroma, has emerged as a promising diagnostic tool. In this study, we aimed to assess the diagnostic performance of ^68^Ga-FAPI-04 PET/CT in HCC patients with and without liver cirrhosis and to explore the relationship between PET metrics, tumor size, and PIVKA-II serum marker. **Methods:** In this prospective single-center study, 59 patients with confirmed HCC (37 with cirrhosis, 22 without) underwent ^68^Ga-FAPI-04 PET/CT. The standard dose (1.5–2.0 MBq/kg) was administered intravenously, and imaging was carried out 60 min post-injection. Semi-quantitative parameters including SUVmax, SUVmean, and tumor-to-background ratio were calculated. Diagnostic performance was assessed using histopathology and multimodal imaging. Statistical analyses included the Mann–Whitney U test and Spearman correlation. **Results:** The overall sensitivity for HCC detection was 89.8%, with a specificity of 60% and accuracy of 87%. Sensitivity and specificity showed a tendency to be lower in cirrhotic compared with non-cirrhotic patients, with a notably higher background liver uptake in cirrhosis (SUVmax 3.60 vs. 1.3, *p* < 0.001), resulting in lower TBR values (3.7 vs. 7.0, *p* < 0.001). A strong correlation between SUVmax and tumor size was seen in non-cirrhotic HCC, while a moderate association between SUVmax and PIVKA-II levels was observed in cirrhotic patients. **Conclusions:** ^68^Ga-FAPI-04 PET/CT demonstrates high sensitivity for HCC detection and may serve as a complementary imaging modality, particularly when interpreted through conventional cross-sectional imaging. Image interpretation in cirrhotic livers may be challenging due to increased background uptake and reduced TBR. Associations between PET-derived parameters, tumor size, and serum PIVKA-II levels should be considered hypothesis-generating and require validation in larger, multicenter studies with clinical outcome data.

## 1. Introduction

Hepatocellular carcinoma (HCC) is the most common primary liver tumor worldwide, accounting for 75–85% of all liver cancer cases [1,2,3]. This malignancy is a leading cause of cancer-related mortality globally [3]. According to GLOBOCAN estimates, in 2022, there were 865.269 newly diagnosed cases of liver cancer worldwide (ranking sixth among all malignancies) and 757.948 deaths (ranking third in overall cancer-related mortality) [2]. Liver cancer occurs most frequently in the Asian region, accounting for 70.1% of all newly diagnosed cases globally. The highest mortality rates are also observed in Asia, where 70% of all liver cancer deaths occur [4]. In Kazakhstan, which is located within the Asian region, liver cancer ranks among the leading oncological diseases in men (fifth place) and occupies a high position in cancer-related mortality (sixth place) in both sexes [4]. The high mortality rate in liver cancer worldwide indicates the limited effectiveness of current strategies for its assessment and treatment [5].

Molecular imaging technologies are rapidly being developed. Positron emission tomography/computed tomography (PET/CT) with various radioactive tracers has opened up new possibilities for noninvasive evaluation of the biological properties of tumors [6]. ^18^F-FDG PET/CT is a classical, standard imaging method for the diagnosis, staging, and monitoring of treatment efficacy in tumors [7,8]. However, researchers agree that the recently introduced radiotracer ^68^Ga-FAPI, applied in hybrid imaging, has several advantages that offer benefits over ^18^F-FDG for HCC imaging [9,10,11,12,13]. Increased background uptake of ^18^F-FDG in liver tissue, as well as well-differentiated HCC lesions with low ^18^F-FDG uptake, may result in an unsatisfactory tumor-to-background ratio (TBR). In contrast, radiolabeled fibroblast activation protein inhibitors (FAPIs) are considered promising agents for the imaging of malignant tumors with a high content of cancer-associated fibroblasts (CAFs), including HCC [14,15,16,17]. Preliminary studies have demonstrated a high expression of fibroblast activation protein (FAP) in HCC [18] and liver cirrhosis [3,7]. Several studies have reported that FAPI provides higher diagnostic value for intrahepatic tumors due to its low background liver uptake [19]; however, cirrhosis may lead to increased background liver uptake of gallium-68, which can affect its diagnostic performance [7,20,21,22,23]. When comparing background liver uptake in relation to cirrhosis status, the reported results are inconsistent: some authors describe higher ^68^Ga-FAPI-04 SUVmax values in patients with cirrhosis and higher TBR values in patients without cirrhosis; other researchers have found that only ^68^Ga-FAPI-04 SUVmean in the liver is significantly higher in cirrhotic patients than in non-cirrhotic ones, whereas another study reports no significant differences in ^68^Ga-FAPI-04 uptake according to liver cirrhosis status [23,24,25]. Therefore, further investigation into the diagnostic potential of ^68^Ga-FAPI-04 PET/CT for HCC according to liver cirrhosis status is warranted.

The need to identify new diagnostic indicators for HCC has led researchers to focus on the serum marker PIVKA-II (a protein induced by vitamin K absence or antagonist II), which shows promising results in HCC, as demonstrated in several scientific studies [26,27].

However, current studies on ^68^Ga-FAPI have mainly focused on its ability to detect hepatic tumors on PET images, whereas the relationship between PET characteristics of HCC and serum PIVKA-II levels, as well as the diagnostic potential of ^68^Ga-FAPI-04 PET/CT for HCC according to liver cirrhosis status, remains insufficiently explored.

Therefore, our aims in this study were to evaluate the diagnostic value of ^68^Ga-FAPI-04 PET/CT for HCC imaging according to liver cirrhosis status and to analyze potential associations between semi-quantitative PET parameters and serum PIVKA-II levels, which may contribute to the development of a more accurate noninvasive approach to liver tumor assessment and a precision medicine method of managing liver cancer.

## 2. Materials and Methods

This single-center prospective observational study was approved by the local ethics committee (No.1641; AR19679719, approval date: 27 September 2023). Written informed consent was obtained from all participants.

**Figure 1:**
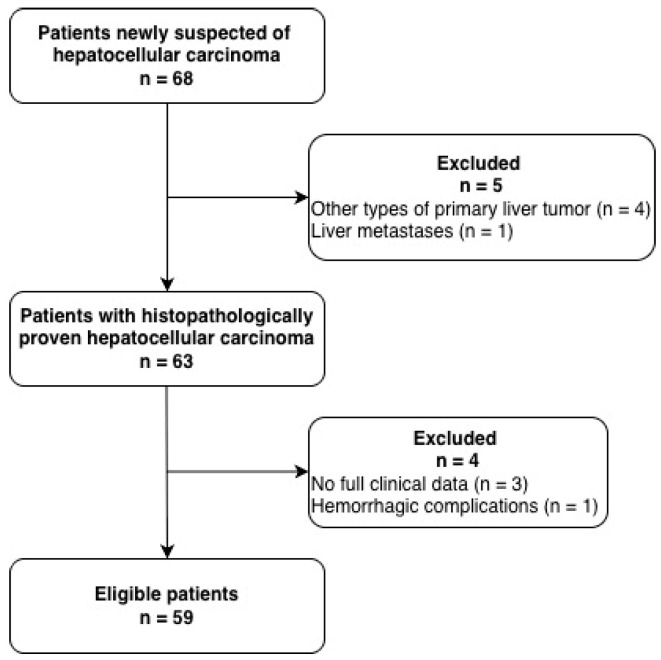
Descriptive flowchart for study.

A total of 68 patients newly suspected of HCC underwent ^68^Ga-FAPI-04 PET/CT. Nine of them were excluded from the study: four were diagnosed with other types of primary liver tumors (three cases of cholangiocarcinoma and one case of neuroendocrine tumor), and one patient had metastatic liver disease; three patients lacked complete clinical data (specifically, no results for the serum marker PIVKA-II), and one patient was documented as having hemorrhagic complications following anticoagulant therapy 3–5 months prior to the PET/CT examination (Figure 1).

A total of 59 patients were included in the study (Me = 61.0 years; range 38–85 years; 45 men and 14 women). All patients were divided into two groups. Group one included 37 patients with HCC associated with liver cirrhosis (Me = 63.0 years; range 42–77 years; 30 men and 7 women). Group two included 22 patients with HCC without cirrhosis (Me = 59.0 years; range, 38–85 years; 15 men and 7 women). The inclusion criteria for both groups were as follows: (a) age ≥ 18 years; (b) undergoing ^68^Ga-FAPI-04 PET/CT; (c) histologically confirmed HCC; (d) measured serum PIVKA-II level; (e) measured serum AFP level; (f) the absence of prior cancer therapy. An additional inclusion criterion for group one was the presence of liver cirrhosis, confirmed by medical history, clinical findings, and imaging methods. An additional inclusion criterion for group two was the absence of liver cirrhosis, confirmed through medical history, clinical findings, and imaging methods. Patients were excluded from the study if they were unsuitable for examination due to pregnancy, had received prior cancer therapy, had an unclear diagnosis of cirrhosis, had hemorrhagic or thrombotic disorders (PTA < 40% or PT-INR ≥ 1.5), or had been receiving anticoagulant therapy within six months prior to the study. Liver cirrhosis was diagnosed according to the recommendations of the American Association for the Study of Liver Diseases (AASLD) and the World Gastroenterology Organization (WGO), including a combination of clinical, laboratory, and imaging findings. The final diagnosis of HCC was confirmed by histopathological examination of liver biopsy specimens or surgical resection samples.

All patients underwent liver ultrasonography, dynamic liver magnetic resonance imaging (MRI), and dynamic liver computed tomography (CT). Histopathological examination, dynamic liver MRI, and dynamic liver CT served as reference standards in assessing the diagnostic value of ^68^Ga-FAPI-04 PET/CT. Serum levels of AFP and PIVKA-II were measured for all patients.

### 2.1. Pathologic Evaluation

Histopathological confirmation was available for all cases of HCC at the patient level: tissue samples were obtained via surgical resection in 35 patients and via biopsy in 24 patients. HCCs were subdivided according to the WHO classification into NOS-HCC or HCC of specific variants [28]. HCCs were assessed on differentiation according to the Edmonson–Steiner grade. At the lesion level, histopathological verification was available for 70 HCC lesions. An additional 28 liver lesions in patients with multiple tumors were not confirmed via histopathology and were classified using typical imaging features on ultrasound, contrast-enhanced CT, and/or contrast-enhanced MRI, as well as follow-up imaging performed after 3–6 months.

Serum levels of AFP and PIVKA-II were analyzed in all patients. Serum PIVKA-II levels were measured using an enzyme-linked immunosorbent assay (ELISA). The reference range for serum PIVKA-II levels was 14–50.8 mAU/mL. Serum AFP levels were measured using an electrochemiluminescence immunoassay analyzer equipped with dedicated reagents, following the manufacturer’s instructions. Serum AFP levels within the range of 0–10 ng/mL were considered to be within the reference interval.

### 2.2. The Procedure of PET/CT Imaging

^68^Ga-FAPI-04 was synthesized and labeled using the Modular Lab-Easy system (Eckert & Ziegler, EZ Medical, Berlin, Germany) following Good Manufacturing Practice (GMP) standards. The radiochemical purity of the solution was >95%. ^68^Ga-FAPI-04 PET/CT scans were performed without requiring any special patient preparation. The radioactivity administered was calculated at a dose of 1.5 to 2.0 MBq per kilogram of patient body weight. Scanning was conducted 60 min post-injection using the Siemens Horizon 3R PET/CT scanner, with a scan range from the orbitomeatal zone to the mid-thigh. CT acquisition parameters included a tube voltage of 130 kV, a tube current of 120 mA, and a slice thickness of 1.5 mm. PET data were collected using a matrix size of 128 × 128 and a 600 mm field of view. Image reconstruction and analysis were performed using Syngo.via post-processing (Siemens Healthineers, Erlangen, Germany).

### 2.3. Evaluation of Images and Lesion-Based Analysis

After PET/CT acquisition, two nuclear medicine physicians, each with more than ten years of experience, independently reviewed all images. Any discrepancies were resolved via a consensus discussion. The classification of additional liver lesions in cases of multiple lesions not confirmed via histopathology was based on prior liver ultrasonography, dynamic liver CT, dynamic liver MRI, and follow-up imaging performed after 3–6 months.

In this study, a lesion-based analysis was performed, with diagnostic parameters evaluated at the level of individual liver lesions. This approach was selected because a proportion of patients presented with multiple focal liver lesions and it allowed for a more detailed characterization of each lesion.

Image interpretation began with the maximum intensity projection (MIP) images in the coronal plane. All images were analyzed in the coronal, axial, and sagittal planes using OsiriX DICOM Viewer, UDI-PI: 14.1.1 (software version).

Radiotracer uptake was considered positive in cases of increased accumulation that could not be associated with physiological biodistribution; other imaging modalities’ results were also taken into account for general assessment. The absence of radiotracer uptake was considered a negative result. The quantitative assessment of radiotracer uptake was automatically provided through standardized uptake values (SUVs) after a three-dimensional region of interest (3D ROI) was selected. The standardized uptake value mean (SUVmean) and standardized uptake value maximum (SUVmax) were measured for normal liver parenchyma; SUVmax and the TBR were measured for primary liver lesions. On the axial PET image, a region of interest (ROI) encompassing the entire lesion was delineated, followed by the calculation of SUVmax. For the calculation of the SUV, circular regions of interest were drawn around the tumor lesions with a focally increased uptake in transaxial slices and automatically adapted to a 3-dimensional volume of interest using analysis software (Siemens Healthineers Full Version: VB60S_HF03) at a 60% isocontour.

Simultaneously, a circular VOI with a volume of 10 mm^3^ was placed within the normal liver parenchyma, at a distance from the lesion, avoiding the course of the hepatic vessels and bile ducts. The mean SUV obtained from this VOI was recorded as the background liver uptake. Ultimately, the TBR of the liver lesion was calculated using the following formula: TBR = SUVmax of liver lesion/SUVmean of background liver uptake. ^68^Ga-FAPI-04-positive lesions were measured on axial PET/CT images through the determination of the longest dimension of tumor lesions with focally increased uptake.

### 2.4. Reference Standard

Histopathological examination served as the standard for diagnosing HCC. In cases where multiple liver lesions were detected on PET/CT images with histopathological confirmation only of the primary tumor node, other lesions were assessed based on liver ultrasonography, dynamic liver CT, dynamic liver MRI, and follow-up imaging performed after 3–6 months. If the final results remained inconclusive, a biopsy of the lesion was conducted followed by histopathological evaluation of the obtained tissue samples.

### 2.5. Statistical Analysis

In this study, we used continuous statistics for semi-quantitative data (SUV and TBR). The distribution of quantitative data was tested for normality using descriptive statistics and the Shapiro–Wilk test for small samples. For asymmetric distributions, the median (Me) and quartiles (Q1 and Q3) were used. Since the data distribution in our study did not follow the normal law, statistical analysis was performed using the nonparametric Mann–Whitney U test. Correlation analysis was carried out through the calculation of the Spearman rank correlation coefficient (r_s_). The critical level of statistical significance (*p*) was assumed to be equal to or less than 0.05. Statistical data analysis was performed using the SPSS 17.0 software package for Windows.

## 3. Results

### 3.1. Patient Characteristics

This study included 59 patients with newly diagnosed HCC (median age, 61.0 years; range, 38–85 years; 45 men and 14 women), divided into two groups: group one, 37 patients with HCC associated with liver cirrhosis (median age, 63.0 years; range, 42–77 years), group two, 22 patients with HCC without cirrhosis (median age, 59.0 years; range, 38–85 years).

All patients underwent liver ultrasonography, dynamic liver MRI, and dynamic liver CT, as well as blood tests to measure serum levels of AFP and PIVKA-II. Histopathological examination, dynamic liver MRI, and dynamic liver CT were used as reference standards in evaluating the diagnostic value of ^68^Ga-FAPI-04 PET/CT.

The final diagnosis of HCC was confirmed via histopathological examination. The diagnosis of liver cirrhosis followed the recommendations of the AASLD and the WGO, incorporating medical history, clinical symptoms, liver function assessment, and imaging for comprehensive evaluation. The clinical characteristics of the patients are summarized in Table 1.

### 3.2. Comparative Analysis of ^68^Ga-FAPI-04 PET/CT Parameters for Visualization of HCC According to the Presence of Liver Cirrhosis

A total of 59 patients with histopathologically confirmed HCC were included in the study, among whom 108 focal liver lesions were identified via imaging methods. As a result, histopathological and imaging findings confirmed that 90.74% (98/108) of the lesions are primary malignant HCC foci: 36 (61.02%) patients had solitary hepatic tumors, and 23 (38.98%) patients had multiple tumors. Among the 108 lesions detected via imaging, 92 liver lesions demonstrated ^68^Ga-FAPI-04 uptake on PET/CT, of which 88 were confirmed as HCC (true-positive lesions) and 4 were determined to be benign liver lesions (false-positive lesions); however, no ^68^Ga-FAPI-04 uptake was detected in 16 liver lesions, of which 10 were confirmed to be HCC lesions (false-negative lesions) and 6 were benign liver lesions (true-negative lesions). The diagnostic performance of ^68^Ga-FAPI-04 PET/CT in HCC evaluation was assessed (Table 2).

As can be seen in Table 2, the diagnostic performance of ^68^Ga-FAPI-04 PET/CT for HCC was as follows: Sn (Sensitivity) = 89.80% (95%CI: 82.03–95.00%); Sp (Specificity) = 60.0% (95%CI: 26.24% to 87.84%); and Acc (Accuracy) = 87.04% (95%CI: 79.21% to 92.73%). For HCC associated with liver cirrhosis, ^68^Ga-FAPI-04 PET/CT demonstrated Sn = 87.30% (95%CI: 76.50% to 94.35%), Sp = 57.14% (95%CI: 18.41% to 90.10%), and Acc = 84.29% (95%CI: 73.62% to 91.89%). For HCC without cirrhosis, the characteristics of ^68^Ga-FAPI-04 PET/CT were as follows: Sn = 94.29% (95%CI: 80.84% to 99.30%), Sp = 66.67% (95%CI: 9.43% to 99.16%), and Acc = 92.11% (95%CI: 78.62% to 98.34%). Analysis of the diagnostic performance of ^68^Ga-FAPI-04 PET/CT according to the liver cirrhosis status showed similar parameters in both groups; however, there was a tendency for the sensitivity, specificity, and accuracy to decrease when imaging HCC lesions in cirrhotic livers.

A total of 88 true-positive tumor lesions on ^68^Ga-FAPI-04 PET/CT were included for further analysis in 59 patients with HCC. Among them, in group one, 55 (62.50%) of HCC lesions were visualized, whereas in group two, 33 (37.50%) of HCC lesions were visualized. Ме SUVmax = 7.05 and Ме TBR = 4.01 were determined for all liver lesions in HCC.

**Figure 2:**
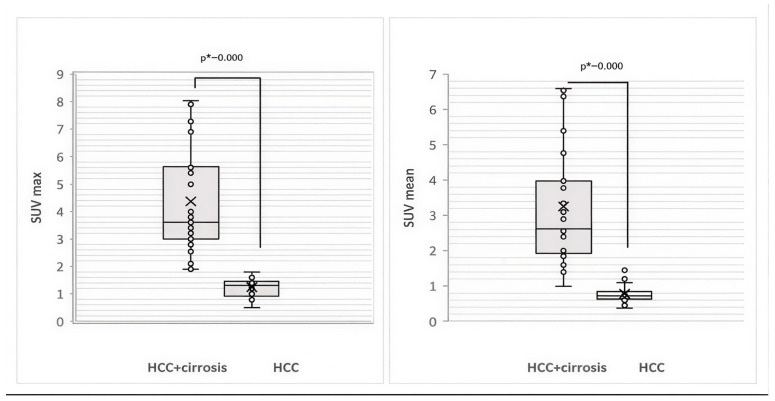
Box plots showing SUVmax and SUVmean of liver uptake of ^68^Ga-FAPI-04 on PET/CT according to liver cirrhosis status in patients with HCC. * Mann–Whitney U test, significance *p*-value < 0.05.

The background liver uptake values of ^68^Ga-FAPI-04 on PET/CT (Me SUVmean and Me SUVmax) were analyzed in patients with HCC according to liver cirrhosis status (Figure 2).

As seen in Figure 2, the two compared groups show a statistically significant difference in the level of liver uptake of ^68^Ga-FAPI: the median values of hepatic SUVmax and SUVmean in HCC associated with cirrhosis are significantly higher than those in HCC without cirrhosis (Ме SUVmax = 3.60 [Q1 = 3.0, Q3 = 5.64] vs. Ме SUVmax = 1.3 [Q1 = 0.92, Q3 = 1.46], *p* < 0.001 [U = 0.0, Z = −6.381]; Ме SUVmean = 2.63 [Q1 = 1.93, Q3 = 3.98] vs. Ме SUVmean = 0.72 [Q1 = 0.63, Q3 = 0.84], *p* < 0.001 [U = 12.0, Z = −6.192]).

**Figure 3:**
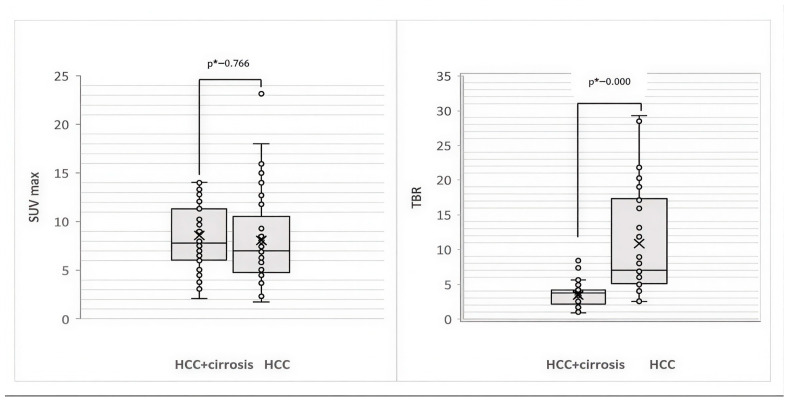
Box plots showing SUVmax and TBR of liver tumors according to liver cirrhosis status in HCC. * Mann–Whitney U test, significance *p*-value < 0.05.

The analysis of SUVmax and TBR values of liver lesions on ^68^Ga-FAPI-04 PET/CT according to liver cirrhosis status is presented in Figure 3.

Comparison of SUVmax values in true-positive liver lesions showed that in HCC associated with cirrhosis, the SUVmax was higher than in HCC without cirrhosis: Ме SUVmax = 7.8 (Q1 = 6.05, Q3 = 11.32) vs. Ме SUVmax = 7.0 (Q1 = 4.77, Q3 = 10.545), *p* = 0.219 (U = 765.000, Z = −1.229). Comparison of TBR values in two groups revealed statistically significantly lower values for HCC associated with cirrhosis than for HCC without cirrhosis: Ме TBR = 3.7 (Q1 = 2.50, Q3 = 4.17) vs. Ме TBR = 7.0 (Q1 = 5.08, Q3 = 17.30), *p* < 0.001 (U = 31.000, Z = −5.894).

**Figure 4:**
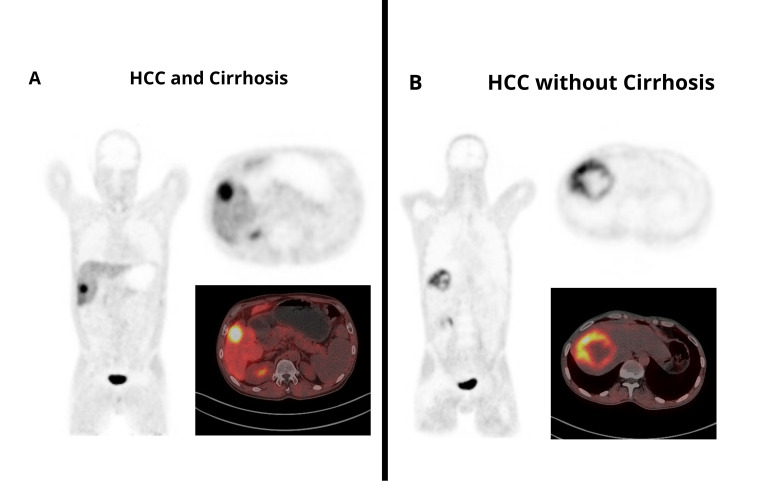
Representative ^68^Ga-FAPI-04 PET/CT images of HCC. (**A**) HCC in a cirrhotic liver, demonstrating increased background hepatic uptake and reduced tumor-to-background contrast. (**B**) HCC in a non-cirrhotic liver, showing lower background uptake and higher TBR.

Thus, ^68^Ga-FAPI-04 PET/CT shows significantly higher background liver uptake in HCC associated with liver cirrhosis than in HCC without cirrhosis; intrahepatic HCC lesions demonstrate higher uptake and a statistically significantly lower TBR for HCC with cirrhosis compared with HCC without cirrhosis. Representative ^68^Ga-FAPI-04 PET/CT images of HCC in cirrhotic and non-cirrhotic livers are shown in Figure 4, illustrating higher background liver uptake and reduced lesion conspicuity in cirrhosis compared with non-cirrhotic livers.

### 3.3. Analysis of the Relationship Between ^68^Ga-FAPI-04 PET/CT Parameters and the Size of HCC Tumor Lesions Associated with Cirrhosis

Based on the results of ^68^Ga-FAPI-04 PET/CT, the median (Me) size of HCC tumor lesions was determined to be Me = 53.0 mm (range, 12.0–139.0). The sizes of HCC tumor lesions were assessed by group: the median tumor size in HCC associated with cirrhosis was Me = 53.0 mm and in HCC without cirrhosis was Me = 50.0 mm.

A correlation analysis using the Spearman correlation coefficient was performed to identify the presence of a relationship between tumor size and PET parameters of hepatic HCC lesions on ^68^Ga-FAPI-04 PET/CT. The calculated Spearman correlation coefficient (r_s_) values are presented in Table 3.

Among all HCC lesions, a moderate positive correlation is found between tumor size and tumor SUVmax r_s_ = 0.504, *p* < 0.001); in the same cohort, no correlation is observed between tumor size and TBR (r_s_ = 0.122, *p* = 0.258).

Correlation analysis between PET parameters on ^68^Ga-FAPI-04 PET/CT and the tumor size of HCC associated with cirrhosis reveals no relationship: the Spearman correlation coefficient between tumor size and tumor SUVmax is r_s_ = 0.207 (*p* = 0.129); the same coefficient between tumor size and TBR is r_s_ = −0.121, (*p* = 0.379).

Among HCC lesions without cirrhosis, a strong positive correlation is found between tumor size and tumor SUVmax (r_s_ = 0.815, *p* < 0.001) and a moderate positive correlation is identified between tumor size and TBR (r_s_ = 0.673, *p* < 0.001).

Thus, based on correlation analysis using the Spearman rank correlation coefficient, a moderate positive relationship is identified between the size of the HCC lesion and the level of ^68^Ga-FAPI-04 uptake. This relationship leads to a strong positive association among HCC lesions without cirrhosis compared with HCC lesions associated with cirrhosis.

### 3.4. Analysis of the Relationship Between PET Parameters of HCC Tumor Lesions on ^68^Ga-FAPI-04 PET/CT and the Serum Marker PIVKA-II Level

To identify the relationship between the serum PIVKA-II level and the ^68^Ga-FAPI-04 PET parameters of HCC lesions, a correlation analysis using calculated Spearman correlation coefficients (r_s_) was performed (Table 4).

Correlation analysis between serum PIVKA-II levels and PET parameters of HCC lesions on ^68^Ga-FAPI-04 PET/CT reveals no relationship between them, either among all HCC lesions (for tumor SUVmax, r_s_ = 0.177, *p* = 0.098; for TBR, r_s_ = 0.128, *p* = 0.234; for tumor size, r_s_ = 0.072, *p* = 0.506) or among HCC lesions without cirrhosis (for tumor SUVmax, r_s_ = −0.087, *p* = 0.632; for TBR, r_s_ = −0.214, *p* = 0.231; for tumor size, r_s_ = −0.320, *p* = 0.070). However, among HCC lesions with cirrhosis, a moderate positive correlation is identified between serum PIVKA-II level and lesion SUVmax (Spearman correlation coefficient r_s_ = 0.438, *p* = 0.001). In addition, a weak positive correlation is observed between serum PIVKA-II level and tumor size in HCC with cirrhosis (Spearman correlation coefficient r_s_ = 0.296, *p* = 0.028).

Thus, using correlation analysis with determination of the nonparametric Spearman rank correlation coefficient, a moderate association can be identified between the serum PIVKA-II level and the degree of ^68^Ga-FAPI-04 uptake in HCC lesions (SUVmax) with cirrhosis. In addition, a weak association is observed between the PIVKA-II level and tumor size. In the group with HCC without cirrhosis, no relationship between the parameters is found.

## 4. Discussion

The classical hybrid imaging method is ^18^F-FDG PET/CT, which is widely used for tumor evaluation [14, 30]. However, according to the European Association for the Study of the Liver (EASL) Clinical Practice Guidelines on the management of hepatocellular carcinoma, ^18^F-FDG uptake was observed in less than 40% of HCC cases [31], limiting the clinical value of this method in HCC imaging [30,31].

Since the first publications on it in 2018–2019 [15,16,22], FAPI PET/CT has attracted increasing attention in oncologic imaging [22,32], becoming an important additional imaging modality. In recent years, the number of studies on the clinical application of FAPI PET/CT has steadily risen [21,32,33], particularly in HCC [3,6,7,20,24,25,34]. This is related to the mechanism of interaction between the FAPI and tumors. FAP is a type II membrane-bound protease capable of remodeling the extracellular matrix by degrading collagen and modifying bioactive signaling peptides in cancer [35]. FAPI, acting as a reagent to target the tumor microenvironment, enables in vivo visualization of CAFs, suggesting promising potential for noninvasive tumor assessment [13,33].

Publications on hybrid imaging with ^68^Ga-FAPI predominantly focus on the diagnostic accuracy of this method in imaging in HCC. This study also assesses the informativeness of the method. The diagnostic performance parameters of ^68^Ga-FAP-04 PET/CT for primary HCC in this work are as follows: sensitivity, 89.80%; specificity, 60.0%; diagnostic accuracy, 87.04%. Our results are comparable to the results of Siripongsatian et al. [5] regarding specificity and Wang et al. [23] (85.7%) or Guo et al. (94%) [24] regarding sensitivity. However, specificity estimates in this work should be interpreted with caution, given the limited number of true-negative lesions and the resulting wide confidence intervals.

The diagnostic performance of ^68^Ga-FAPI-04 PET/CT in our study was lower than that reported by Shi et al., who found 100% sensitivity and specificity [25]. Similarly, Siripongsatian et al. described 100% sensitivity but a lower specificity of 75%, attributed to two false-positive cases [5]. Interesting findings are described in a study where the authors used ^18^F-FAPI-42 and report 96% sensitivity, 58.3% specificity, and 83.8% accuracy in primary liver tumors imaging—higher in sensitivity but lower in specificity and accuracy than our results [36]. Liu et al., in a meta-analysis, concluded that the sensitivity of FAPI PET/CT for primary liver tumor imaging ranges from approximately 87% to 100% [37], correlating with our findings. FAPI PET/CT is characterized by high sensitivity in HCC detection. However, the relatively limited specificity observed in our study indicates that ^68^Ga-FAPI-04 PET/CT should be considered a complementary imaging modality rather than a standalone diagnostic tool for HCC.

Approximately 80–90% of HCC cases develop in patients with liver cirrhosis, and survival improves with early HCC diagnosis [31]. Therefore, one focus of our study was to investigate the diagnostic value of ^68^Ga-FAPI-04 PET/CT in primary HCC associated with cirrhosis. Considering the limited number of studies dedicated to this topic and the fact that existing publications mainly evaluate the informativeness of this method for primary liver tumors in general rather than HCC alone [11], our study analyzed the diagnostic performance of ^68^Ga-FAPI-04 PET/CT for primary HCC according to liver cirrhosis status. The results of our study show equivalent findings for HCC associated with cirrhosis (sensitivity 87.30%, specificity 57.14%, diagnostic accuracy 84.29%) compared to HCC without cirrhosis (sensitivity 94.29%, specificity 66.67%, diagnostic accuracy 92.11%), with a tendency to decline in HCC associated with cirrhosis. Peng et al. also reported similar sensitivities for ^68^Ga-FAPI PET/CT in detecting intrahepatic malignancies with and without cirrhosis, at 93% and 98%, respectively [11], with a similar reduction in sensitivity in the presence of cirrhosis. Analysis of background liver uptake of ^68^Ga-FAPI-04 on PET/CT in our study revealed median liver uptake values of Me SUVmax = 3.60 and Me SUVmean = 2.63 in cirrhotic livers, compared to Me SUVmax = 1.3 and Me SUVmean = 0.72 in non-cirrhotic livers. In their literature review, Manupela et al. highlight the conflicting evidence in the existing published data regarding background liver uptake in relation to cirrhosis [19]. In our study, background liver uptake of ^68^Ga-FAPI-04 was consistently higher in cirrhotic livers compared to non-cirrhotic livers, which resulted in reduced lesion conspicuity and lower TBRs in this group. This finding may be explained by increased FAP expression in perisinusoidal cells within regenerative nodules and fibrotic septa [38]. Moreover, FAP is a cell-associated dipeptidyl peptidase and gelatinase with dual specificity, expressed by activated hepatic stellate cells involved in tissue remodeling during cirrhosis [38]. FAP may facilitate extracellular matrix remodeling mediated by hepatic stellate cells, and its expression during cirrhosis correlates with the severity of liver fibrosis [3,38]. Peng et al. obtained results similar to our findings [11]. Guo et al. reported higher liver SUVmax on ^68^Ga-FAPI-04 PET/CT in primary liver tumors associated with liver cirrhosis (*p* < 0.01), suggesting that concomitant cirrhosis may potentially complicate the interpretation of FAPI PET/CT [24]. In another study [23], only SUVmean values were significantly higher in liver tumors associated with liver cirrhosis compared to those without cirrhosis (*p* < 0.001). Shi et al. [25] found no significant differences in ^68^Ga-FAPI-04 liver uptake but demonstrated that ^68^Ga-FAPI-04 uptake is associated with FAP expression in malignant liver tumors. According to Yang et al., background liver uptake is higher in advanced liver fibrosis compared to mild fibrosis and an increased number of FAP-expressing fibroblasts correlates with histological fibrosis severity [13]. Subsequently, in this study, SUVmax in true-positive HCC lesions and TBR were analyzed according to liver cirrhosis status. The results show a slightly higher SUVmax in HCC associated with cirrhosis than in HCC without cirrhosis: Me SUVmax = 7.8 versus Me SUVmax = 7.0, *p* = 0.219; comparative analysis of TBR between groups reveals that TBR is significantly lower in HCC associated with cirrhosis than without cirrhosis: Me TBR = 3.7 versus Me TBR = 7.0, *p* < 0.001. Similar findings were reported by Peng et al., who studied these parameters in malignant liver lesions [11]. Guo et al. described higher TBR for HCC without cirrhosis, but this result was described as nonsignificant [24]. Another study also reports higher TBR for HCC without cirrhosis, which is noted as nonsignificant (*p* = 0.300) compared to TBR in HCC associated with cirrhosis [23]. Despite conflicting data across studies, most authors agree that ^68^Ga-FAPI PET/CT demonstrates high sensitivity for HCC detection. Background liver uptake is higher in cirrhotic livers, resulting in lower TBR values in HCC associated with cirrhosis compared to non-cirrhotic HCC. However, opinions diverge regarding decreasing performance in ^68^Ga-FAPI PET/CT image interpretation in HCC associated with cirrhosis. Our study’s analysis of the diagnostic performance of ^68^Ga-FAPI-04 PET/CT for primary HCC revealed equivalent outcomes for HCC associated with cirrhosis compared to HCC without cirrhosis, with a tendency to decrease in informativeness in detecting HCC in cirrhotic livers; background liver uptake values are higher for HCC associated with cirrhosis than for without (*p* < 0.001), while TBR is lower for HCC associated with cirrhosis compared to HCC without cirrhosis (*p* < 0.001). Despite the positive results regarding diagnostic capabilities reported in the existing literature, the clinical application of FAPI PET/CT is limited due to false-positive and false-negative findings [6,19]. In our study, based on imaging and pathomorphological analysis, four false-positive lesions (two dysplastic nodules and two local inflammatory dilations of peripheral intrahepatic bile ducts) and ten false-negative liver lesions were identified. For two dysplastic nodules demonstrating false-positive ^68^Ga-FAPI-04 uptake in our study, a biopsy with subsequent histopathological examination was required. In contrast, localized inflammatory dilatation of peripheral intrahepatic bile ducts was clearly differentiated on CT/MRI images. Therefore, in clinical practice, the accurate characterization of liver lesions requires the correlation of FAPI PET/CT findings with contrast-enhanced CT/MRI. False-positive ^68^Ga-FAPI uptake has been reported in various benign liver conditions, including adenomas, hemangiomas, dysplastic nodules, inflammatory lesions, fibrosis, and cirrhosis [6,21,23,25,39,40]. Wang et al. associated increased ^68^Ga-FAPI-04 uptake with more advanced fibrosis inside or within false-positive lesions presented as angiomyolipoma, fibrous nodular hyperplasia, and inflammatory nodules [23]. False-positive results lower the specificity of FAPI PET/CT in diagnosing primary liver tumors, including HCC, representing a potential risk of misdiagnosis [6]. Therefore, caution is recommended when interpreting intrahepatic lesions with increased ^68^Ga-FAPI-04 uptake as malignant [23], a conclusion supported by our study. Additionally, false-positive findings related to prior therapeutic interventions in the liver have been reported, leading authors to suggest that the method suffers from low specificity in differentiating residual or recurrent tumor disease from post-radiation, surgical, or interventional inflammatory reactions [39], a conclusion confirmed by other studies [6]. Non-specific fibrosis caused by postoperative inflammation may also contribute to positive ^68^Ga-FAPI-04 uptake [40]. Therefore, increased ^68^Ga-FAPI-04 uptake in cirrhotic livers should be interpreted in conjunction with MRI/CT findings and the clinical context, with follow-up or biopsy recommended in equivocal cases. False-negative results can generally be explained by higher background FAPI uptake in cirrhotic livers [19]. Researchers have confirmed that false-negatives are associated with smaller HCC lesion sizes in cirrhotic livers and the degree of tumor tissue hypoxia [23]. Hypoxia may rationally explain the positive correlation between ^68^Ga-FAPI-04 uptake intensity and tumor size [23]. Other authors have suggested that the precise mechanisms underlying false-negative FAPI results remain unclear and may relate to heterogeneous FAPI expression within tumors and complex interactions with the tumor microenvironment [6]. For example, Liang et al. identified 6 false-negative HCC lesions among 39 malignant tumors [6]. Similar findings were reported by Wang et al., with 5 out of 35 HCC lesions being negative on ^68^Ga-FAPI-04 PET/CT [23], corresponding to our own data (10 out of 98 HCC lesions showed false-negative results). Hence, the use of FAPI PET/CT for HCC detection may be limited by false-positive and false-negative results, as confirmed in this study; accumulated scientific evidence suggests that false-negative results are associated with both heterogeneous FAPI expression in primary HCC lesions and high background liver uptake in cirrhotic livers, while the exact mechanisms behind false-negatives remain incompletely understood.

In this study, we analyzed the correlation between ^68^Ga-FAPI-04 PET parameters and HCC tumor size. Overall, tracer uptake tended to increase with larger tumor size, whereas TBR did not show a consistent association across all lesions. When stratified by liver status, tracer uptake and tumor size were primarily associated in non-cirrhotic HCC, while no clear relationship between PET parameters and tumor size was observed in cirrhotic HCC. Similar associations between SUVmax, TBR, and tumor size in ^68^Ga-FAPI-04 PET/CT-positive liver lesions have been reported previously [23]. These findings are consistent with our results for HCC lesions without cirrhosis. Prior studies have also demonstrated that ^68^Ga-FAPI-04 PET/CT may be more sensitive than ^18^F-FDG PET/CT in the detection of small HCC lesions (≤2 cm diameter) [23]. Tumor size is known to correlate with malignancy in primary liver tumors [31]. Based on our results and the literature data, we hypothesize that higher ^68^Ga-FAPI-04 uptake may reflect more aggressive tumor biology, particularly in non-cirrhotic HCC. Moreover, better visualization of HCC (i.e., higher TBR), correlating with larger tumor size, is observed in non-cirrhotic livers. AFP has long been used as a standard biomarker for HCC and is considered more objective than imaging alone [26]. However, its diagnostic sensitivity and specificity, particularly in early-stage disease, remain limited [29,31]. In this context, PIVKA-II may serve as an additional tumor marker for HCC, complementing AFP in diagnosis and in the assessment of treatment efficacy [27].

Thus, in this study, we aimed to correlate ^68^Ga-FAPI-04 PET parameters with serum PIVKA-II levels in HCC. Spearman correlation analysis shows no association between ^68^Ga-FAPI-04 PET parameters and serum PIVKA-II levels for either all HCC lesions or HCC without cirrhosis. However, in HCC associated with cirrhosis, a moderate positive correlation is found between tumor SUVmax and serum PIVKA-II level. This may be explained by the activation, proliferation, and accumulation of fibroblasts, associated with aggressive tumor behavior in its microenvironment, being more advanced in cirrhosis [3]. Furthermore, FAP expression is activated in hepatic stellate cells during cirrhosis and correlates with fibrosis severity [3]. Previous studies have reported that serum PIVKA-II levels increase in association with HCC complications and are linked to poorer prognosis [27]. Therefore, the observed associations between tumor tracer uptake and PIVKA-II levels should be interpreted cautiously and considered hypothesis-generating rather than definitive indicators of prognosis. Additionally, a weak positive correlation was observed between tumor size and serum PIVKA-II in cirrhotic HCC lesions. The literature suggests that PIVKA-II levels correlate positively with tumor size on imaging, indicating their role in prognosticating disease severity [27], consistent with our findings showing this association in cirrhotic HCC. Moreover, elevated PIVKA-II concentrations are linked to biologically aggressive HCC and poor prognosis [27,31]. Thus, this study’s correlation analysis results highlight that in cirrhotic HCC, ^68^Ga-FAPI-04 uptake and tumor size, combined with high PIVKA-II levels, are of prognostic significance in relation to fibrosis severity and worse outcomes in this patient group. Therefore, we hypothesize that in HCC associated with liver cirrhosis, the degree of ^68^Ga-FAPI-04 uptake and tumor size, in combination with elevated serum PIVKA-II levels, may reflect underlying tumor–stroma interactions and potentially be of prognostic relevance. However, the observed relationships represent correlational associations only and should be considered hypothesis-generating. Further studies in larger, preferably multicenter cohorts with outcome data are required to validate any prognostic implications.

This study has limitations. Firstly, the sample size is limited: a larger number of observations is recommended for the group with HCC without cirrhosis. Secondly, diagnostic performance was evaluated on a lesion-based rather than a patient-based level. This approach may lead to the overestimation of sensitivity due to the clustering of multiple lesions within individual patients and may limit the direct translation of the results to patient-level clinical decision-making. Thirdly, inter-reader agreement was not formally assessed using quantitative metrics (e.g., kappa or intraclass correlation coefficients). Fourthly, the study lacked a control group of cirrhotic patients without HCC, which limits the interpretation of background liver uptake and specificity estimates in cirrhotic livers. Lastly, the study design is limited by being single-center. The limited number of published data on ^68^Ga-FAPI PET/CT for detecting HCC according to liver cirrhosis status requires further investigation in a larger cohort, including a representative control group of patients with liver cirrhosis. The absence of previous studies exploring the relationship between ^68^Ga-FAPI PET/CT parameters and serum PIVKA-II levels in HCC shows the need for further research on a larger cohort to improve noninvasive diagnosis of HCC.

The study results demonstrate that ^68^Ga-FAPI-04 PET/CT can be used as an additional imaging method for HCC, underscoring its value for primary malignant liver lesions. One of the most significant advantages of gallium-68-based radiopharmaceuticals (FAPIs) is their potential in theranostic applications. FAPI-based imaging can be transformed into targeted radionuclide therapy [41]. Given our findings, ^68^Ga-FAPI-04 PET/CT may facilitate the selection of patients with HCC for theranostics based on molecular and metabolic information.

## 5. Conclusions

^68^Ga-FAPI-04 PET/CT demonstrated high sensitivity and clinical relevance in imaging HCC. However, the relatively low specificity observed in this study indicates that ^68^Ga-FAPI-04 PET/CT should be considered a complementary imaging modality rather than a standalone diagnostic tool for HCC.

The diagnostic performance of ^68^Ga-FAPI-04 PET/CT was comparable in HCC associated with liver cirrhosis and in non-cirrhotic HCC, with a tendency toward reduced diagnostic accuracy in cirrhotic livers. The background liver uptake of ^68^Ga-FAPI-04 was significantly higher and TBR was significantly lower in cirrhotic livers, which may complicate image interpretation. Better lesion visualization, correlating with larger tumor size, was observed in non-cirrhotic livers.

In HCC associated with liver cirrhosis, the degree of tumor ^68^Ga-FAPI-04 uptake and tumor size, in combination with elevated serum PIVKA-II levels, may reflect underlying tumor–stroma interactions and represent hypothesis-generating findings with potential prognostic relevance. These observations require validation in larger cohorts with outcome data. The observed association between FAPI uptake and PIVKA-II levels highlights the potential clinical significance of this approach and supports further investigations into FAPI-based imaging, including possible implications for anti-FAP-targeted theranostic strategies.

## Figures and Tables

**Table 1:** Clinical characteristics of patients.

Total Number of Patients	59
Median, age (y, range)	61.0 (range 38–85)
Gender	
Male	45 (76.27%)
Female	14 (23.73%)
Groups	
Group one: HCC and cirrhosis	37 (62.71%)
Group two: HCC	22 (37.29%)
Liver tumor lesions	
Number of patients/solitary tumor	36/36 (61.02%)
Number of patients/multiple tumor lesions	23/62 (38.98%)
Child–Pugh–Turcotte score for group one	
Class A	15 (40.54%)
Class B	18 (48.65%)
Class С	4 (10.81%)
MELD score [1] (*Me* = 12)	
≤12	14
>12	19
Child–Pugh–Turcotte/MELD [29]	
CPT Class A: MELD score, median, (score, range)	8 (range 6–16)
CPT Class B: MELD score, median, (score, range)	15 (range 12–21)
Histological types of HCC [28]	
NOS-HCC	49 (83.05%)
HCC of specific variants	
SH-HCC	9 (15.25%)
Fibrolamellar carcinoma	1 (1.70%)
Differentiation	
G1	10 (16.95%)
G2	42 (71.19%)
G3	7 (11.86%)
Differentiation for group	
G1: Group one/group two	6 (16.22%)/4 (18.18%)
G2: Group one/group two	26 (70.27%)/16 (72.73%)
G3: Group one/group two	5 (13.51%)/2 (9.09%)
HBs Ag (+)	44 (74.58%)
Serum tumor markers	
AFP > 10.0 ng/mL	54 (91.53%)
PIVKA-II > 50.8 mAU/mL	52 (88.14%)
Blood transaminases	
For men ALT > 45 U/L, for women ALT > 35 U/L	42 (71.19%)
For men AST > 45 U/L, for women AST > 35 U/L	46 (77.97%)
Metastatic HCC	6 (10.17%)

y, years; HCC, hepatocellular carcinoma; MELD, Model for End-Stage Liver Disease; CPT, Child–Pugh–Turcotte; NOS-HCC, not otherwise specified hepatocellular carcinoma; SH-HCC, steatohepatitic hepatocellular carcinoma; G1, well-differentiated HCC; G2, moderately differentiated HCC; G3, poorly differentiated HCC; HBs Ag, hepatitis B surface antigen; AFP, alpha-fetoprotein; PIVKA-II, protein induced by vitamin K absence or antagonist II; ALT, alanine aminotransferase; AST, aspartate aminotransferase.

**Table 2:** Diagnostic performance of ^68^Ga-FAPI-04 PET/CT in HCC evaluation.

Characteristics	HCC		HCC and Cirrhosis		HCC Without Cirrhosis	
	D “+”	D “-”	D “+”	D “-”	D “+”	D “-”
Positive	TP = 88	FP = 4	TP = 55	FP = 3	TP = 33	FP = 1
Negative	FN = 10	TN = 6	FN = 8	TN = 4	FN = 2	TN = 2
Accuracy/Acc, % (95%CI)	87.04 (79.21–92.73)		84.29 (73.62–91.89)		92.11 (78.62–98.34)	
Sensitivity/Sn, % (95%CI)	89.80 (82.03–95.00)		87.30 (76.50–94.35)		94.29 (80.84–99.30)	
Specificity/Sp, % (95%CI)	60.0 (26.24–87.84)		57.14 (18.41–90.10)		66.67 (9.43–99.16)	

HCC, hepatocellular carcinoma; D “+”, disease “+”; D “-”, disease “-”; 95%CI, 95% confidence interval; TP, true-positive; FN, false-negative; FP, false-positive; TN, true-negative.

**Table 3:** The calculated Spearman correlation coefficients between PET parameters and tumor size in HCC lesions on ^68^Ga-FAPI-04 PET/CT.

PET Parameters	The Calculated Spearman Correlation Coefficient, r_s_	*p*-Value
All lesions, n = 88		
SUVmax of lesion	0.504	0.000 *
TBR	0.122	0.258
Group one: HCC and cirrhosis, n = 55		
SUVmax of lesion	0.207	0.129
TBR	−0.121	0.379
Group two: HCC without cirrhosis, n = 33		
SUVmax of lesion	0.815	0.000 *
TBR	0.673	0.000 *

*, *p*-value < 0.001.

**Table 4:** Calculated Spearman correlation coefficients between serum PIVKA-II level and PET parameters of HCC tumor lesions on ^68^Ga-FAPI-04 PET/CT.

PET Parameters	Calculated Spearman Correlation Coefficient, r_s_	*p*-Value
All lesions, n = 88		
SUVmax of lesion	0.177	0.098
TBR	0.128	0.234
Size of lesion	0.072	0.506
Group one: HCC and cirrhosis, n = 55		
SUVmax of lesion	0.438	0.001 **
TBR	0.105	0.444
Size of lesion	0.296	0.028 *
Group two: HCC without cirrhosis, n = 33		
SUVmax of lesion	−0.087	0.632
TBR	−0.214	0.231
Size of lesion	−0.320	0.070

*, *p*-value ≤ 0.05. **, *p*-value < 0.01.

## Data Availability

The datasets generated during the current study, as well as the raw data, are available from the corresponding author on reasonable request.

## Abbreviations

The following abbreviations are used in this manuscript:

AASLD	American Association for the Study of the Liver Diseases
AFP	alpha-fetoprotein
ALT	alanine aminotransferase
AST	aspartate aminotransferase
CAF	cancer-associated fibroblasts
CPT	Child–Pugh–Turcotte
CT	computed tomography
DICOM	Digital Imaging and Communications in Medicine
EASL	European Association for the Study of the Liver
ELISA	enzyme-linked immunosorbent assay
FAP	fibroblast activation protein
FAPI	fibroblast activation protein inhibitor
FDG	fluorodeoxyglucose
FN	false-negative
FP	false-positive
GMP	Good Manufacturing Practice
HCC	hepatocellular carcinoma
HBs Ag	hepatitis B surface antigen
MELD	Model for End-Stage Liver Disease
MIP	maximum intensity projection
MRI	magnetic resonance imaging
PET/CT	positron emission tomography/computed tomography
PIVKA-II	protein induced by vitamin K absence or antagonist II
PT-INR	prothrombin time—international normalized ratio
PTA	prothrombin time activity
ROI	region of interest
rs	Spearman rank correlation coefficient
SUV	standardized uptake value
SUVmax	maximum standardized uptake value
SUVmean	mean standardized uptake value
TBR	tumor-to-background ratio
TN	true-negative
TP	true-positive
UL	upper limit
VOI	volume of interest
WGO	World Gastroenterology Organization
